# Precocious and Early Central Puberty in Children With Pre-existing Medical Conditions: A Single Center Study

**DOI:** 10.3389/fped.2019.00035

**Published:** 2019-02-14

**Authors:** Sarah Winter, Adélaïde Durand, Raja Brauner

**Affiliations:** Fondation Ophtalmologique Adolphe de Rothschild and Université Paris Descartes, Paris, France

**Keywords:** central precocious puberty, chromosomal duplication, early puberty, epilepsy, hypothalamic-pituitary-gonadal axis, precocious puberty, psychomotor delay, psychiatric disorders

## Abstract

**Background:** Precocious and early puberty are reported findings in children with pre-existing medical conditions including certain syndromes. Series pertaining to such situations are limited.

**Methods:** A retrospective, single-center study was conducted on children with central precocious puberty (onset before the age of 8 years in girls and 9 years in boys) or early puberty (onset between 8 and 9 years in girls and between 9 and 10.5 years in boys) diagnosed on the background of a known pre-existing chronic significant medical condition. Patients with a CNS tumor and those exposed to cranial irradiation were excluded.

**Results:** Precocious puberty was diagnosed in 13 patients and early puberty in 12. Mean age at onset of puberty was 6.65 ± 2.3 years in girls (*n* = 15) and 9.4 ± 0.84 years in boys (*n* = 10). The most common disorders were psychomotor delay (*n* = 12), psychiatric disorders (*n* = 7) and/or epilepsy (*n* = 5). Precocious or early puberty was among the symptoms experienced by patients with a variety of syndromes including lipofuscinosis (2 siblings), Dravet syndrome and Silver-Russel syndrome. Pituitary stalk interruption with agenesis of olfactory bulbs and optic nerve atrophy was found on imaging in one patient who presented with blindness, epilepsy, and autism spectrum disorder. The other diseases associated with precocious or early puberty are adrenocorticotropic deficiency, dyspraxia and bone abnormalities, glomerulopathy with complete renal failure, and repeated intra-fetal deaths in the mother. Karyotype analysis revealed chromosomal duplication (chromosome 15 in 2 cases; chromosomes 17 and 11 in one case each) in 4 of 8 patients evaluated.

**Conclusions:** Data from patients with complex disease who experience precocious or early puberty may provide clues regarding the genetic determinants of pubertal development.

## Introduction

The onset of puberty is driven by an increase in amplitude and frequency of gonadotropin-releasing hormone (GnRH) pulses after a quiescent period during childhood. The reactivation of pulsatile GnRH secretion leads to increases in the secretion of the gonadotropins, luteinizing hormone (LH), and follicle-stimulating hormone (FSH) by the anterior pituitary gland and consequent activation of gonadal function (1). Patients experiencing pubertal development as a result of the activation of the hypothalamic-pituitary-gonadal axis before the ages of 8 years (girls) or 9 years (boys) are diagnosed with central precocious puberty (CPP) (2). Those experiencing this activation between the ages of 8–9 years (girls) (3) and 9–10.5 years (boys) (4) are known to have early puberty, an entity that is frequently considered as a normal variant and yet may underscore significant disease.

Complex interactions among genetic, nutritional, environmental, and socio-economic factors control the onset of normal puberty (5, 6). In a previous report on 154 patients from 139 families presenting with precocious or early puberty, we were able to demonstrate that the mode of inheritance of either phenotype is predominantly maternal (7). More than half of the families had individuals with either precocious or early puberty, suggesting that both entities may be controlled by similar factors and could likely be studied as a single entity in this particular context (7).

Precocious or early puberty have also been reported in the context of a variety of pre-existing medical conditions including certain syndromes. Series on this particular presentation were absent until the recent presentation of Canton et al. (8) including 26 patients (representing 17% of their idiopathic CPP) presenting as CPP and at least two additional features and/or conditions, characterizing complex phenotypes. The presence of a known, pre-existing, medical diagnosis provides the opportunity to further study potentially contributing factors to precocious or early puberty and shed additional light on the mechanisms of pubertal onset in this population. To this end, we have summarized in this report clinical, laboratory, imaging, and cytogenetic data from 25 patients presenting with this association. Patients with a central nervous system tumor and those exposed to cranial irradiation were excluded from this study.

## Subjects and Methods

### Ethics Approval and Consent to Participate

The protocol was approved by the Ethical Review Committee Comité de Protection des Personnes Ile de France III (certificate AC 048). All of the clinical investigations were conducted according to the principles expressed in the Declaration of Helsinki. Written informed consent for the evaluations was obtained from the children's parents and was included in their hospital medical records. No additional activity to routine patient care was performed. The authors had no direct interaction with the patients enrolled in the study, except for their medical follow-up, which was performed by R. Brauner.

### Patients

This retrospective, single-center, cohort study included all patients monitored for precocious or early puberty in the context of a pre-existing chronic significant medical condition by a senior pediatric endocrinologist (R. Brauner) in a university pediatric hospital between 2004 and 2014. In girls, CPP was diagnosed by breast development before the age of 8 years accompanied by the presence of pubic or axillary hair, a growth rate >2 SD scores (SDS) the year before clinical evaluation and/or a bone age (BA) >2 years above chronological age (6). In boys, CPP was diagnosed by testicular enlargement (testicular volume index >3.5–4 cm^2^) before the age of 9 years (2). Early puberty was diagnosed by the appearance of these signs between the ages of 8 and 9 years in girls (3) and 9 and 10.5 years in boys (4). A total of 15 girls and 10 boys fulfilled the diagnostic criteria to be included in this study.

### Methods

The initial evaluation included the determination of birth data, height expressed as SDS, growth rate, weight, body mass index (BMI) (expressed as SDS), pubertal stage rated according to *Marshall and Tanner* and BA assessed using the *Greulich and Pyle* method (9–11) except in 7 (28%) patients. Age at the first pubertal sign was reported by the parents.

The GnRH test, consisting of the intravenous injection of GnRH (100 μg/m^2^) and measurements of LH and FSH peaks at 0, 30, 60, and 90 min, was performed in 9 girls and 9 boys. Plasma LH, FSH, estradiol and testosterone concentrations were measured using radioimmunoassay. The following values were considered to be pubertal: LH/FSH peak ratio after GnRH test ≥ 0.66 in girls and ≥ 2 in boys (12), plasma estradiol concentrations ≥ 15 pg/mL in girls and testosterone levels ≥ 0.5 ng/mL in boys. Pelvic ultrasound evaluation was performed in 7 girls to evaluate the estrogenic stimulation of the uterus and to determine GnRH analog treatment. A uterus length value ≥35 mm was considered to be pubertal (13). Magnetic resonance imaging (MRI) was performed to exclude hypothalamic-pituitary lesion. It was not performed in 3 girls with CPP because they had a normal neurological evaluation, were >6 years of age at the onset of puberty and had low plasma estradiol concentration (< 15 pg/mL, cases 6 and 8) or because MRI has already been performed in the brother (case 9). Plasma 17-hydroxyprogesterone and testosterone concentrations were measured in all of the boys and in the girls with pubic or axillary hair development as the first pubertal sign to exclude abnormal androgen secretion and congenital adrenal hyperplasia (14). Plasma thyroxin and thyroid-stimulating hormone concentrations were measured to exclude hypothyroidism, and 24-h urinary cortisol levels were measured to exclude hypercortisolism in those who were overweight or had a rapid weight increase; all of the values were normal. Karyotype analysis was performed in 6 girls and 2 boys in the medical follow-up of their pre-existing medical condition.

The results are expressed as the means ± *SD*.

## Results

### Characteristics of the Patients

The characteristics of the 25 patients at the initial evaluation are shown in Table 1. Fifteen girls and 10 boys were included in this study, corresponding to a sex ratio at 1.5. Mean age at onset of puberty was 6.65 ± 2.3 years in girls and 9.4 ± 0.84 years in boys. In girls, the age at onset of puberty was < 3 years in 2 patients (13.3%), 3–7 years in 4 patients (26.6%), 7–8 years in 4 patients (26.6%), and 8–9 years in 5 patients (33.3%). In boys, the age at onset of puberty was 8–9 years in 3 patients (30.0%) and 9–10.5 years in 7 patients (70.0%). Thus, 66.5% of the girls and 30% of the boys have CPP, the others having early puberty. LH/FSH peak ratio was pubertal in 6 girls (66.6% of those evaluated) and in 7 boys (77.8%). Bone age was advanced over chronological age by 0.5 ± 0 years in girls and 0.85 ± 0.9 years in boys. One girl (case 1) experienced the onset of breast and pubic hair development at the age of 0.5 years. Pubertal development remained stable without treatment until the age of 8 years when breast and plasma estradiol concentration increased. Five girls (30.0%) and six boys (60.0%) received GnRH agonist treatment.

**Table 1:** Characteristics of the patients with precocious or early puberty associated with other disorders.

	**Age at onset, years**	**Age at evaluation, years**	**Bone age, years**	**Tanner stage**	**Testicular volume, cm^**2**^**	**Height at evaluation, SDS**	**BMI, SDS**	**LH/FSH peak ratio**	**Estradiol, pg/mL (girls) or testosterone, ng/mL (boys)**	**Treatment**
**GIRLS**										
1	0.5	0.8	1.3	B2P2		0.0	0.0	0.1	0	No
2	2.5	2.7	ND	B2P1		0.0	−1.0	2.5	33	Yes
3	4.9	5.0	ND	B2P1		+4.0	+2.0	0.4	2	No
4	6.2	6.3	8.3	B3P1		+2.0	0.0	0.7	21	Yes
5	6.5	9.3	12.0	B5P5		+3.0	+4.0	ND	ND	No
6	6.8	7.8	7.4	B2P2		+1.0	+1.5	0.7	< 20	No
7	7.0	7.2	9.5	B2P1		+3.5	+2.0	0.3	0	No
8	7.0	9.0	10.0	B3P3		0.0	+1.0	ND	10	Yes
9	7.5	7.8	ND	B2P2		−0.5	−3.0	2.2	4	Yes
10	7.5	9.9	8.9	B2P1		+0.5	−2.0	0.9	2	No
11	8.0	8.8	11.0	B3P2		+1.5	+1.0	9.9	177	Yes
12	8.5	9.5	10.0	B3		0.0	+1.0	ND	5	No
13	8.8	9.2	ND	B2P1		+1.0	+0.5	ND	ND	No
14	9.0	10.0	ND	B4P3		+2.0	+2.5	ND	ND	No
15	9.0	10.5	ND	B4P4		−0.5	+1.5	ND	26	No
**BOYS**										
16	8.0	12.0	13.5	P3	15.0	+2.0	+1.0	4.0	9.2	No
17	8.3	8.5	8.0	ND	6.0	0.0	−2.0	6.9	4.7	Yes
18	8.4	8.4	7.0	ND	4.0	0.0	0.0	6.0	0.3	Yes
19	9.1	9.4	ND	P3	8.0	+1.5	+1.5	2.9	1.6	Yes
20	9.4	11.6	14.0	ND	8.0	+2.5	−1.0	1.6	2.4	Yes
21	9.5	10.5	12.0	P2	ND	+2.0	+1.0	ND	1.9	No
22	10.0	11.0	12.0	P4	13.5	+1.5	+1.0	5.0	1.2	No
23	10.1	10.3	13.0	P3	9.0	−1.0	0.0	30.0	0.9	Yes
24	10.4	10.7	11.5	P3	6.0	−1.0	−2.5	0.8	0.9	No
25	10.4	10.8	11.0	P5	14.0	+1.0	0.0	6.4	3.1	Yes

*ND, not determined; SDS, standard deviation score*.

Consanguinity was reported in one case (case 2) without any additional details. A history of familial precocious or early puberty was reported in 8 cases (5 girls and 3 boys); it affected the mother in 6 cases and an older sister in 2 cases (Table 2).

**Table 2:** Clinical, karyotype, and MRI findings associated with precocious or early puberty.

	**Familial history of early puberty**	**Clinical features**	**Karyotype**	**MRI**
**GIRLS**				
1	No	Psychomotor delay, microcephaly	Normal	Normal
2	No	Psychomotor delay, pectus excavatum	Normal	Normal
3	No	Psychomotor delay, hyperactivity, achromic skin lesions, ovary hernia	17p13.3 duplication	Normal
4	No	Psychomotor delay, epilepsy, pervasive mental disorders	Normal	Normal
5	No	Autism	ND	Slight cerebellar atrophy
6	Yes	4 intra-fœtal deaths in the mother	ND	ND
7	Yes	Bipolar disorder	ND	Pituitary microadenoma
8	Yes	Autism	Normal	ND
9	No	Lipofuscinosis	ND	ND
10	No	Anorexia	ND	Normal
11	No	Dravet syndrome, dorsal scoliosis	ND	Normal
12	No	Psychomotor delay	Chromosome 15 duplication	ND
13	No	Epilepsy	ND	Normal
14	Yes	Autism	ND	ND
15	Yes	Psychomotor delay, microcephaly	ND	Normal
**BOYS**				
16	Yes	Epilepsy, hyperactivity	ND	Normal
17	No	Lipofuscinosis	ND	Normal
18	No	Psychomotor delay, epilepsy, autism, blindness	Chromosome 11 duplication	PSIS, agenesis of the olfactory bulbs and optic nerve atrophy
19	No	Psychomotor delay, microcephaly, dismorphy (thumb hypoplasia, syndactyly, simple ear)	ND	Normal
20	No	Adrenocorticorticotrophin deficiency	ND	Normal
21	Yes	Psychomotor delay	Chromosome 15 duplication	Normal
22	No	Epilepsy, dyspraxia,	ND	Normal
23	No	Dyspraxia, bones abnormalities (irregular epiphysis, brachymesophalangy)	ND	Normal
24	Yes	Silver Russel syndrome, hypospadias	ND	Normal
25	No	Glomerulopathy, complete renal failure	ND	Normal

*ND, not determined; MRI, magnetic resonance imaging; PSIS, pituitary stalk interruption syndrome*.

### Pre-existing Medical Diagnoses Associated With Precocious or Early Puberty

In all of the patients, the diagnosis of the associated disorder was made before that of precocious or early puberty, except in case 25 with glomerulopathy and complete renal failure that required renal transplantation, who was diagnosed during the evaluation of early puberty by systematic urinary analysis.

Clinical, MRI and cytogenetic (karyotype) findings are detailed in Table 2. The most common associated medical diagnoses were psychomotor delay (12 cases) and/or epilepsy (5 cases). Syndromes included lipofuscinosis (diagnosed in one brother and his sister; cases 9 and 17), Dravet syndrome (case 11), and Silver Russel syndrome (case 24). Seven patients had psychiatric diagnoses including autism spectrum disorder (cases 5, 8, 14, and 18), pervasive developmental disorders (case 4), bipolar disease (case 7), and eating disorder (case 10). In one patient with blindness, psychomotor delay, epilepsy, and autism (case 18), the cerebral MRI performed because of multiple hypothalamic-pituitary deficiencies showed pituitary stalk interruption syndrome with agenesis of the olfactory bulbs and optic nerve atrophy. The other conditions associated with precocious or early puberty were adrenocorticotropic hormone deficiency (case 20), dyspraxia and bone abnormalities (case 23), and glomerulopathy with complete renal failure (case 25). The remaining patient (case 6) had no known pre-existing medical diagnosis but her mother experienced 4 pregnancies complicated by 4 intra-fetal deaths in the mother.

Karyotype analysis revealed abnormalities in 4 of the 8 patients in whom these data were available. These were chromosomal duplications (chromosome 15 in 2 cases, 17 and 11 in one case each). All patients with chromosomal abnormalities had psychomotor delay.

## Discussion

Despite the data suggesting that age at the onset of puberty is primarily driven by genetic factors, the genetic determinants of the timing of pubertal development and particularly of precocious or early puberty remain unknown in a majority of cases (15–17).

In our best knowledge, only one multicentric series of 26 patients presenting as CPP and at least two additional features and/or conditions has been reported in 2018 as an Abstract by Canto et al. (8). They represent 17% of their idiopathic CPP, while the girls of the present study represent 4.5% (10/219) of those seen for CPP in the same conditions. Their most clinical features were: overweight or obesity (*n* = 15), born small for gestational age (*n* = 10), learning difficulties/intellectual disability/autistic spectrum (*n* = 7), short stature (*n* = 7), motor and/or speech delay (*n* = 6), high palate (*n* = 6), acanthosis nigricans (*n* = 5), and hyperinsulinemia (*n* = 5). Five patients were previously diagnosed with Temple syndrome (uniparental disomy of chromosome 14), Williams syndrome (7q11.23 microdeletion) or deletions in SHOX (*n* = 3).

Our study findings suggest that the association between precocious or early puberty and a variety of pre-existing medical conditions may provide additional insight. Indeed, out of 8 patients presenting with psychomotor delay on whom cytogenetic data were available, 50% had chromosomal duplications. Extensive efforts have been made to elucidate the mechanisms that reactivate pulsatile GnRH secretion at the time of puberty, and several genes have been identified in the complex network of inhibitory, stimulatory and permissive neuroendocrine factors involved in the control of puberty onset (Table 3). In the last decade, the kisspeptin system was revealed as a major gatekeeper of puberty onset (55). Several loss-of-function mutations in *KISS1R* were described in patients with congenital isolated hypogonadotropic hypogonadism (56). Only two mutations related to this pathway—one mutation in the gene encoding kisspeptin-1 and one in the gene encoding its receptor—have been associated with CPP (18, 19). In 2012, Tusset et al. identified a variant in the *TAC3* gene in a Brazilian girl with CPP (20). In 2013, applying whole exome sequencing, Abreu et al. were able to identify 4 loss-of-function mutations in *MKRN3* (encoding makorin RING-finger protein 3), an imprinted gene located on the long arm of chromosome 15q11.2, in five of 15 families with CPP (21). Later, Macedo et al. and Settas et al. reported novel *MKRN3* heterozygous mutations, and currently 10 different loss-of-function mutations of *MKRN3* have been described (22, 57). Interestingly, this gene is located in the Prader-Willi syndrome critical region; patients with Prader-Willi syndrome typically have incomplete sexual development; however, few cases of children with CPP have been reported (31–34).

**Table 3:** Reported data related to genetic factors and various disorders associated with precocious or early puberty.

**Gene, chromosomal abnormality or clinical association**	**Primary findings**	**Authors (Ref)**
**GENES**		
*KISS1* or *KISS1R* (*GPR54*)	CPP and autosomal dominant GPR54 mutation (p.A386P) (1 girl) CPP and *KISS1* mutations: p.P74S mutation in a heterozygous state (1 boy) and pPH90D in a homozygous state (2 unrelated girls)	Teles et al. (18) Silveira et al. (19)
*TAC3*	CPP caused by a heterozygous mutation p.A63P (1 girl)	Tusset et al. (20)
*MKRN3*	CPP caused by mutations in the imprinted gene MRKN3; paternally inherited	Abreu et al. (21) Macedo et al. (22)
**CHROMOSOMAL ABNORMALITIES**		
Deletion of 9p	CPP and mental retardation, dysmorphy (2 girls, 1 boy)	Funderburk, (23) Eshel et al. (24) Cisternino et al. (25)
1p36 deletion syndrome	CPP and mental retardation, epilepsy, growth delay, congenital heart defects, characteristic facial appearance (1 girl)	Kurosawa et al. (26)
46XX/69XXX mixoploidy	CPP and central hypothyroidism (1 girl aged 5 months)	Jäverlä et al. (27)
Triple X syndromeDuplication of chromosome 15Duplication of chromosome 9	CPP and mental retardation in all patients (2 girls, 1 boy)	Grosso et al. (28)
Inv dup (15) syndrome	CPP and mental retardation, epilepsy, behavioral problems, malformations (2 girls of 10 patients)	Grosso et al. (29)
Maternal uniparental disomy for chromosome 14	CPP and growth retardation, hypotonia, scoliosis (1 boy)	Temple et al. (30)
**SYNDROMES AND/OR MEDICAL ASSOCIATIONS**		
Prader-Willi syndrome	CPP in patients with Prader-Willi syndrome (1 boy) and growth hormone deficiency (1 boy), treated with GnRH (1 girl), and growth hormone (1 girl)	Vanelli et al. (31) Pusz et Rotenstein, (32) Crino et al. (33) Lee et Hwang, (34)
Kabuki syndrome	CPP and lower lip pits (1 patient) or epilepsy, polymicrogyria, and nephrotic syndrome (1 girl)	Franceschini et al. (35) Di Gennaro et al. (36)
Epidermal nevus syndrome	CPP and epidermal nevus syndrome (1) and hypophosphatemic vitamin D-resistant rickets (1 girl)	Tay et al. (37) Ivker et al. (38)
Cohen syndrome	CPP in identical female twins	North et al. (39)
Williams syndrome (WS)	CPP in 18.3% of girls (of 171 girls)	Partsch et al. (40)
Angelman syndrome (AS)	Premature thelarche (2 girls)	Young et al. (41) Katzos et al. (42)
Aicardi syndrome	CPP in 42% of the girls	Glasmacher et al. (43)
Floating-Harbor syndrome (FHS)	CPP and growth hormone deficiency	Stagi et al. (44)
Cardio-facio-cutaneous syndrome	CPP in 1 case	Celik et al. (45)
PEHO syndrome	CPP and progressive encephalopathy, hypsarrhythmia, and optic atrophy syndrome	Alfadhel et al. (46)
Lipofuscinosis	CPP in a girl with late infantile form	Aysun et al. (47)
Dravet syndrome	CPP and deletion involving SCN1A and SMEI (1 girl)	Madia et al. (48)
Rett syndrome	CPP and deceleration of head growth, hypotonia, respiratory alkalosis (1 girl)	Bas et al. (49) Holm, (50)
Neonatal encephalopathy	Early puberty in 7 (4.2%) of 161 girls	Robertson et al. (51)
*NRXN1* deletion	CPP and early onset developmental delay, epilepsy, gastroesophageal reflux (2 sisters)	Harrison et al. (52)
Neurological disorders	Electroencephalographic tracing abnormalities in 81% of patients with CPP	Liu et al. (53)
Epilepsy	CPP and dysmorphy, hypotonia, and seizures; 3 siblings	Smith et al. (54)

Chromosomal abnormalities were found in 4 patients (among 8 evaluated) of 25 (16%) of the present series: chromosome duplication of 11, 15 (*n* = 2) and 17. Cases of precocious or early puberty associated with chromosomal abnormalities have been reported (Table 3): deletion (23–25) or duplication (28) of 9p, deletion of 1p36 (26), 46,XX/69XXX mixoploidy (27), triple X syndrome (28), duplication or inversion duplication of 15 (29), or maternal uniparental disomy for chromosome 14 (30). These data suggested that performing a karyotype analysis should be recommended for patients with precocious or early puberty and associated disorders without any obvious etiology (primarily tumor or neurofibromatosis).

The association of precocious or early puberty with syndromes and/or medical history is not uncommon and, in most of the cases, the causative factor for this association is unknown. It has been reported in patients with Kabuki syndrome, epidermal nevus syndrome, Cohen syndrome, Williams syndrome, Angelman syndrome, Aicardi syndrome, Floating-Harbor syndrome, cardio-facio-cutaneous syndrome or PEHO syndrome (35–46). In our series, two siblings diagnosed with lipofuscinosis developed CPP. In the literature, this association has been described in one case report (47) in which the authors suggested that the early onset of puberty could be attributed to selective destruction of inhibitory tracts within the hypothalamus, chronic disruption of GnRH containing neurons or stimulation of gonadotropin release from the pituitary by the accumulation of lipofuscin-like material. CPP has also been associated with neurologic disorders, such as Dravet syndrome, Rett syndrome, neonatal encephalopathy or epilepsy (48–54). In our series, neurological and/or psychiatric disorders affected most of the patients. This observation is consistent with the findings reported in the literature; in nearly all of the chromosomal abnormalities or the syndromes mentioned above, the patients suffered from neurological disorders, including psychomotor delay, epilepsy, and encephalopathy. Epilepsy and CPP may also co-occur in the context of a central nervous system tumor or insult (58) which should be ruled out via appropriate imaging (59). There have been studies suggesting that valproic acid or other antiepileptic agents, such as clonazepam, could contribute to precocious or early puberty; so far, no evidence could be found in the literature (60–62). A lot of patients in the present study received various seizure medications.

## Limitations and Strengths

The 25 patients constitute a small series. However, they were collected over 10 years by one senior pediatric endocrinologist, and no other series had been reported until that of the Sao Paulo University hospital group who reported recently 26 patients (8). We included patients with precocious or early puberty because, as reported in the familial forms, including our series (7), the families frequently include both entities suggesting that they may be controlled by similar factors. Only a small number of patients had a karyotype. The patients of this series are followed by other pediatric specialist, mainly neurologist who posed the indication of the karyotype and sent the patients to us because their pubertal development began early. The Sao Paulo group reported only two patients evaluated for karyotype (See above) (8). Furthermore, as most of the patients were referred to us by a pediatric neurologist, that could explain why so many of the patients had psychomotor delay and/or epilepsy. This could affect the generalizability of the findings of the present study.

## Conclusion

Although our cohort included a small number of patients, this study is the first, to our knowledge, to describe in detail a series of patients with precocious or early puberty occurring in the context of a pre-existing medical diagnosis. The majority of patients included in this study presented neurological and/or psychiatric disorders, some fulfilling the diagnostic criteria of a syndrome, and others not. Surprisingly high rates of chromosomal abnormalities were found in assessed patients. Additional studies are needed to investigate the mechanisms through which precocious or early puberty is triggered in patients with complex medical histories.

## Author Contributions

SW collected and analyzed the data and prepared the manuscript. AD collected and analyzed the data. RB directed the work, participated to the preparation and edited the manuscript according to the questions of the Reviewers. All authors discussed the results, commented on the manuscript, and approved the final version to be submitted.

### Conflict of Interest Statement

The authors declare that the research was conducted in the absence of any commercial or financial relationships that could be construed as a potential conflict of interest.

## Abbreviations

BMI: body mass index
BA: bone age
CPP: central precocious puberty
FSH: follicle-stimulating hormone
GnRH: gonadotropin-releasing hormone
LH: luteinizing hormone
MRI: magnetic resonance imaging
SDS: standard deviation score.
